# Forkhead box transcription factor L2 activates *Fcp3C* to regulate insect chorion formation

**DOI:** 10.1098/rsob.170061

**Published:** 2017-06-14

**Authors:** Yu-Xuan Ye, Peng-Lu Pan, Ji-Yu Xu, Zhang-Fei Shen, Dong Kang, Jia-Bao Lu, Qing-Lin Hu, Hai-Jian Huang, Yi-Han Lou, Nai-Ming Zhou, Chuan-Xi Zhang

**Affiliations:** 1 State Key Laboratory of Rice Biology and Ministry of Agriculture Key Laboratory of Molecular Biology of Crop Pathogens and Insects, Institute of Insect Science, Zhejiang University, Hangzhou 310058, People's Republic of China; 2 College of life Science, Zhejiang University, Hangzhou 310058, People's Republic of China

**Keywords:** forkhead box transcription factor l2, follicle cell protein 3C, follicular cells, chorion formation, female infertility

## Abstract

Most animals are oviparous. However, the genes regulating egg shell formation remain not very clear. In this study, we found that *Nilaparvata lugens* Forkhead box transcription factor L2 (*Nl*FoxL2) directly activated follicle cell protein 3C (*NlFcp3C*) to regulate chorion formation. *NlFoxL2* and *NlFcp3C* had a similar expression pattern, both highly expressed in the follicular cells of female adults. Knockdown of *NlFoxL2* or *NlFcp3C* also resulted in the same phenotypes: obesity and female infertility. RNA interference (RNAi) results suggested that *NlFcp3C* is a downstream gene of *NlFoxL2*. Furthermore, transient expression showed that *Nl*FoxL2 could directly activate the *NlFcp3C* promoter. These results suggest that *NlFcp3C* is a direct target gene of *Nl*FoxL2. Depletion of *NlFoxL2* or *NlFcp3C* prevented normal chorion formation. Our results first revealed the functions of Fcp3C and FoxL2 in regulation of oocyte maturation in an oviparous animal.

## 1. Introduction

High fecundity based on oogenesis is a typical characteristic of most insects and is also the major cause of pest outbreaks. Oogenesis can be subdivided into three broad developmental periods: pre-vitellogenesis, vitellogenesis and choriogenesis. The chorion in insect eggs is usually synthesized by the follicular cells and carries out the essential function of protecting the embryo from external agents during development [1,2]. The vitelline membrane (VM) is the first eggshell layer to be synthesized at the end of the vitellogenesis period, dependent upon 20-hydroxyecdysone signalling [3]. After this step, chorionic layers (wax layer, innermost chorionic layer, endochorion and exochorion) are initiated during the choriogenesis period [4]. Chorion genes can be divided into six gene families: ErA/ErB, A/B and HcA/HcB genes are expressed during early, middle and late choriogenesis, respectively [5]. In *Bombyx mori*, CCAAT/enhancer-binding proteins (C/EBPs) regulate early and middle chorion genes, while transcription factors of the GATAβ family are responsible for late chorion gene expression [6,7]. Despite this accumulating wealth of knowledge, whether other signal pathways and transcript factors also regulate insect egg shell formation remains unclear.

Forkhead box L2 (FoxL2) is a member of the winged helix/forkhead transcription factor family, which has a remarkable functional diversity and is involved in a wide variety of biological processes [8]. In humans, FoxL2 is essential for granulosa cell differentiation and ovary maintenance [9]. Approximately 97% of adult-type granulosa cell tumours of the ovary harbour a missense point mutation in the *FoxL2* gene [10,11]. *FoxL2* heterozygous mutations result in blepharophimosis ptosis epicanthus inversus syndrome, which is characterized by eyelid abnormalities often associated with premature ovarian failure [12–15]. In mice, homozygous *FoxL2* mutations resulted in the absence of secondary follicles and oocyte atresia [16]. *FoxL2* is also required to prevent the transdifferentiation of an adult ovary to a testis [17]. In addition to humans and mice, *FoxL2* has also been studied in numerous species such as fishes, chickens, frogs and goats [18]. In the pea aphid *Acyrthosiphon pisum*, RNA-seq data revealed that *FoxL2* is predominantly expressed in sexual females [18]. In the yellow fever mosquito, *Aedes aegypti*, the transcript level of *FoxL2* in ovaries was elevated after a blood meal. Knockdown of *FoxL2* in *Ae. aegypti* resulted in a significant reduction in deposited eggs. It has been suggested that *FoxL2* may be involved in the regulation of mosquito reproduction [19]. However, the actual functions of *FoxL2* in ovipara are still not well understood.

To better understand the gene regulating egg shell formation in ovipara and the functions of *FoxL2* and its potential application in insect pest control, we used the brown planthopper (BPH) *Nilaparvata lugens* Stål (Hemiptera: Delphacidae), one of the most destructive insect pests of rice crops [20], as a model to explore the potential transcriptional targets of *FoxL2* and their functions. After silencing a wide range of ovary-specific genes, respectively, in BPH, we found that knockdown of *NlFoxL2* or *NlFcp3C* resulted in the same phenotypes (obesity and female infertility), which arouses our interest in the relationship between these two genes and their functions in egg maturation.

## 2. Results

### 2.1. Sequence analysis

**Figure 1.**
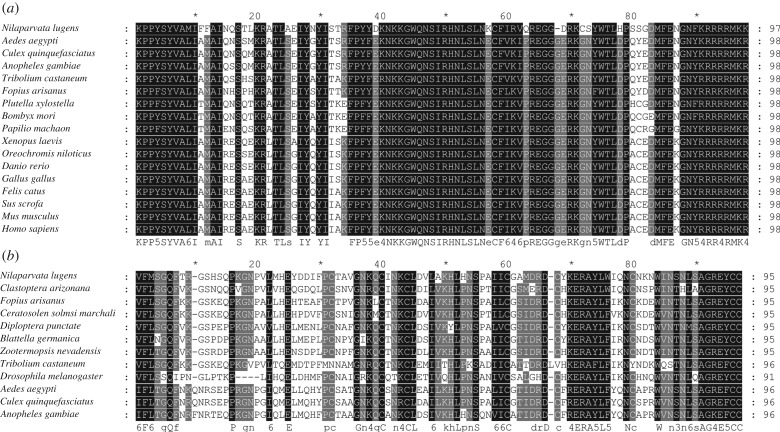
Alignment of amino acid sequences of FoxL2 and Fcp3C. (*a*) *Nl*FoxL2 was aligned with FoxL2 orthologues from 16 other species; (*b*) *Nl*Fcp3C was aligned with Fcp3C orthologues from 11 other species. The alignments were performed using the ClustalX program.

The complete ORF sequence of *NlFoxL2* is 1254 bp in length and has the potential to encode a 417 amino acid residue peptide containing a typical Foxhead domain (97 amino acid DNA-binding domain). Multiple alignments of FoxL2 orthologues from 17 species using the ClustalX program showed that FoxL2 was highly conserved from invertebrates to vertebrates (figure 1*a*). The ORF of *NlFcp3C* is 528 bp long and has the potential to encode a 175 amino acid residue peptide. BLAST analysis for *Nl*Fcp3C in NCBI revealed that Fcp3C orthologues exist only in insects. Multiple sequence alignments of the orthologues from 12 species revealed that Fcp3C was well conserved in insects (figure 1*b*).

### 2.2. Developmental expression patterns

**Figure 2.**
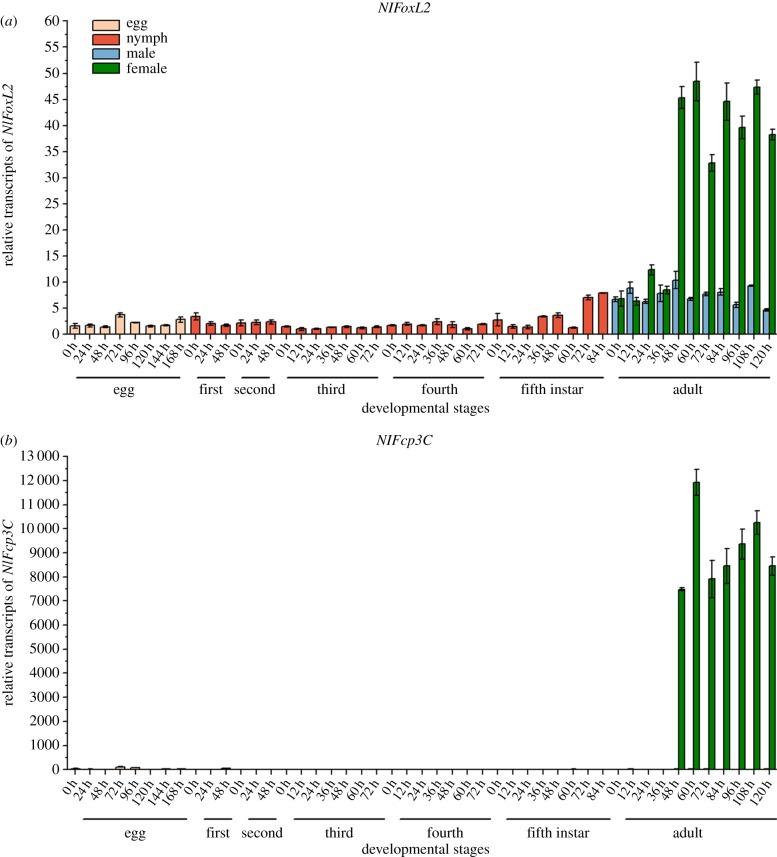
Expression of *NlFoxL2* and *NlFcp3C* in different developmental stages of *Nilaparvata lugens* by qPCR. (*a*) Developmental expression patterns of *NlFoxL2*; (*b*) Developmental expression patterns of *NlFcp3C*. Each total RNA sample was extracted from whole insects at all life stages of BPHs (*n* = 200 eggs, *n* = 30 nymphs for five different instars, respectively; *n* = 30 female adults and male adults, respectively). Samples were collected every 12 or 24 h from the very beginning of each stage. *Nl18S* was used as an internal control gene. Values are means ± standard error of the mean (s.e.m.) from three experiments.

The developmental expression patterns of *NlFoxL2* and *NlFcp3C* were assessed by real-time qPCR. Total RNA was extracted from various developmental samples containing all life stages of BPHs (eggs, nymphs in five different instars, female adults and male adults). The results showed that *NlFoxL2* and *NlFcp3C* transcripts had similar expression patterns, with transcripts peaking approximately 48 h after female adult eclosion and then being maintained at a high level thereafter. The transcripts of *NlFoxL2* in eggs, nymphs and male adults were maintained at a relatively low level, while *NlFcp3C* was expressed nearly exclusively in female adults (figure 2). As ovaries usually reach maturity 2–3 days after female emergence, the expression profiles of *NlFoxL2* and *NlFcp3C* were in line with ovary development.

### 2.3. Tissue-specific expression patterns

**Figure 3.**
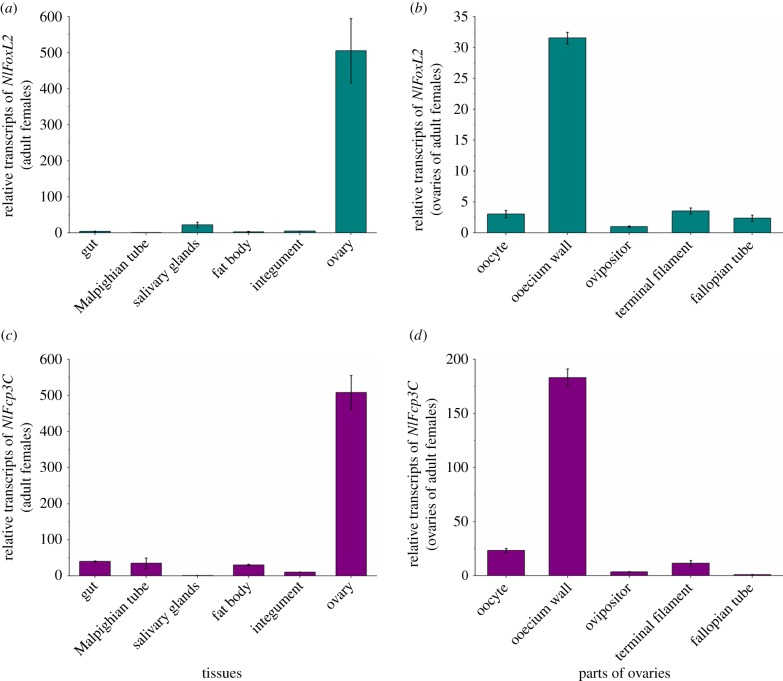
Expression of *NlFoxL2* and *NlFcp3C* in different tissues of *Nilaparvata lugens* by qPCR. (*a*) Expression of *NlFoxL2* in different tissues; (*b*) expression of *NlFoxL2* in different parts of the ovaries; (*c*) expression of *NlFcp3C* in different tissues; (*d*) expression of *NlFcp3C* in different parts of the ovaries. Each total RNA sample used for (*a*) and (*c*) was extracted from different tissues including the gut, Malpighian tube, salivary glands, fat body, integument and ovary, which were dissected from 50 female adults 48–72 h after eclosion. Each total RNA sample used for (*b*) and (*d*) was extracted from different parts of the telotrophic meroistic ovaries of 50 female adults 48–72 h after eclosion including oocyte, ooecium wall, ovipositor, terminal filament and fallopian tube. *Nl18S* was used as an internal control gene. Values are means ± s.e.m. from three experiments.

The tissue-specific expression patterns of *NlFoxL2* and *NlFcp3C* were analysed using real-time qPCR in six tissues of the female *N. lugens* (48–72 h after adult emergence): integument, gut, Malpighian tube, fat body, salivary gland and ovary. Both *NlFoxL2* and *NlFcp3C* were mainly expressed in the ovaries (figure 3*a*,*c*), suggesting specific functions for these genes in female reproduction. To further stratify the gene expression in the ovaries, total RNAs isolated from different parts of ovaries, including oocyte, ooecium wall, ovipositor, terminal filament and fallopian tube, were used for the qPCR analyses. The results showed that both *NlFoxL2* and *NlFcp3C* were highly expressed in the ooecium wall, which mainly contains follicular cells (figure 3*b*,*c*).

### 2.4. Phenotypes of RNA interference

**Figure 4.**
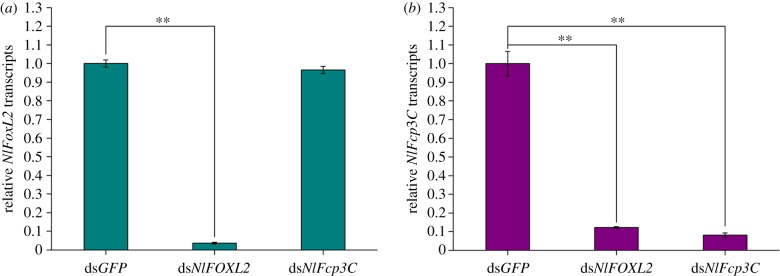
RNAi efficiencies of *NlFoxL2* and *NlFcp3C* and influences on each other. (*a*) Relative transcripts of *NlFoxL2*; (*b*) relative transcripts of *NlFcp3C*. dsRNA (50 ng per insect) for *NlFoxL2* or *NlFcp3C* was injected into newly emerged female adults (within 2 h). RNAi efficiency was investigated using real-time qPCR. Each total RNA sample for these two genes was extracted from 10 BPHs 3 days after injection. ds*GFP* was injected as negative control for the non-specific effects of dsRNA. Values are means ± s.e.m. from three experiments. **p < 0.01 (Student's *t*-test), difference from ds*GFP*.

**Figure 5.**
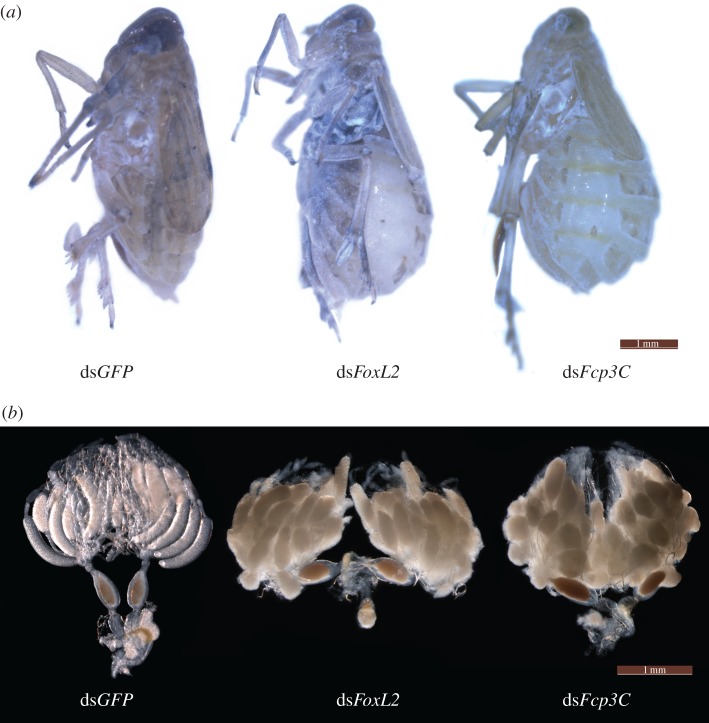
Phenotypes of insects injected with dsRNA for *NlFoxL2* or *NlFcp3C.* (*a*) Severely obese female adults 5 days after injection; (*b*) ovaries dissected from female adults 5 days after injection. dsRNA (50 ng per insect) for *NlFoxL2 or NlFcp3C* was injected into newly emerged female adults (within 2 h). ds*GFP* was injected as negative control for the non-specific effects of dsRNA.

**Figure 6.**
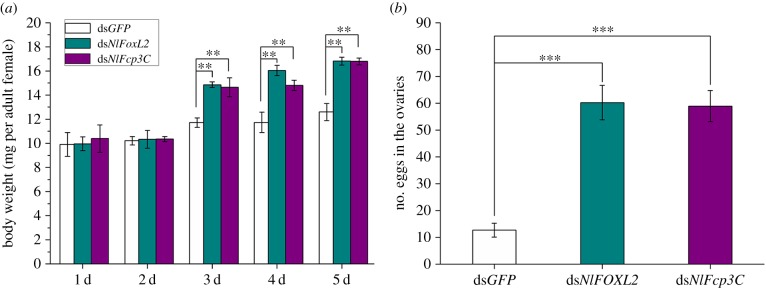
Knockdown of *NlFoxL2* or *NlFcp3C* increased body weight and number of eggs. (*a*) Body weights of 20 female adults 1–5 days after injection, respectively; (*b*) numbers of eggs in the ovaries 5 days after injection. dsRNA (50 ng per insect) for *NlFoxL2 or NlFcp3C* was injected into newly emerged female adults (within 2 h). ds*GFP* was injected as negative control for the non-specific effects of dsRNA. Values are means ± s.e.m. from three experiments. **p < 0.01 and ***p < 0.001 (Student's *t*-test), difference from ds*GFP*.

To determine the possible functions of *NlFoxL2* and *NlFcp3C*, double-stranded RNAs (dsRNAs) targeting each of these genes were injected into newly emerged female adults (within 2 h). Real-time qPCR analysis showed that each dsRNA efficiently suppressed the transcript levels of their target genes 3 days after injection (figure 4). Starting from the third day after injection, the body size of the insects injected with dsRNA targeting *NlFoxL2* or *NlFcp3C* became substantially larger than that of the insects in the ds*GFP* group, with lateral membranes of each segment and inter-segmental membranes in the abdomen obviously stretched, indicating obesity (figure 5*a*). The average weight of the BPHs injected with ds*NlFoxL2* or ds*NlFcp3C* was significantly heavier than that of the ds*GFP* group from the third day after injection. On the fifth day, BPHs knocked down for *NlFoxL2* or *NlFcp3C* were approximately 33% heavier than those in the ds*GFP* group (figure 6*a*).

Every ovariole on BPHs injected with only ds*GFP* for 5 days contained one or, at most, two fully developed banana-shaped oocytes. However, knockdown of *NlFoxL2* or *NlFcp3C* resulted in oocyte dysplasia. Upon dissection of the ovaries, no fully developed oocytes were observed on the fifth day after injection of ds*NlFoxL2* or ds*NlFcp3C*: the short oocytes formed near-spherical shapes rather than the normal banana shapes (figure 5*b*). As a result, female BPHs treated with ds*NlFoxL2* or ds*NlFcp3C* were unable to lay eggs, though the number of oocytes longer than 0.4 mm in the ovaries greatly increased (figure 6*b*). A large accumulation of the abnormal oocytes eventually led to an increase in the ovarian volume (figure 5*b*). However, abnormalities were not observed in nymphs and male adults injected with either ds*NlFoxL2* or ds*NlFcp3C*.

### 2.5. Depletion of *NlFoxL2* or *NlFcp3C* prevented chorion formation

**Figure 7.**
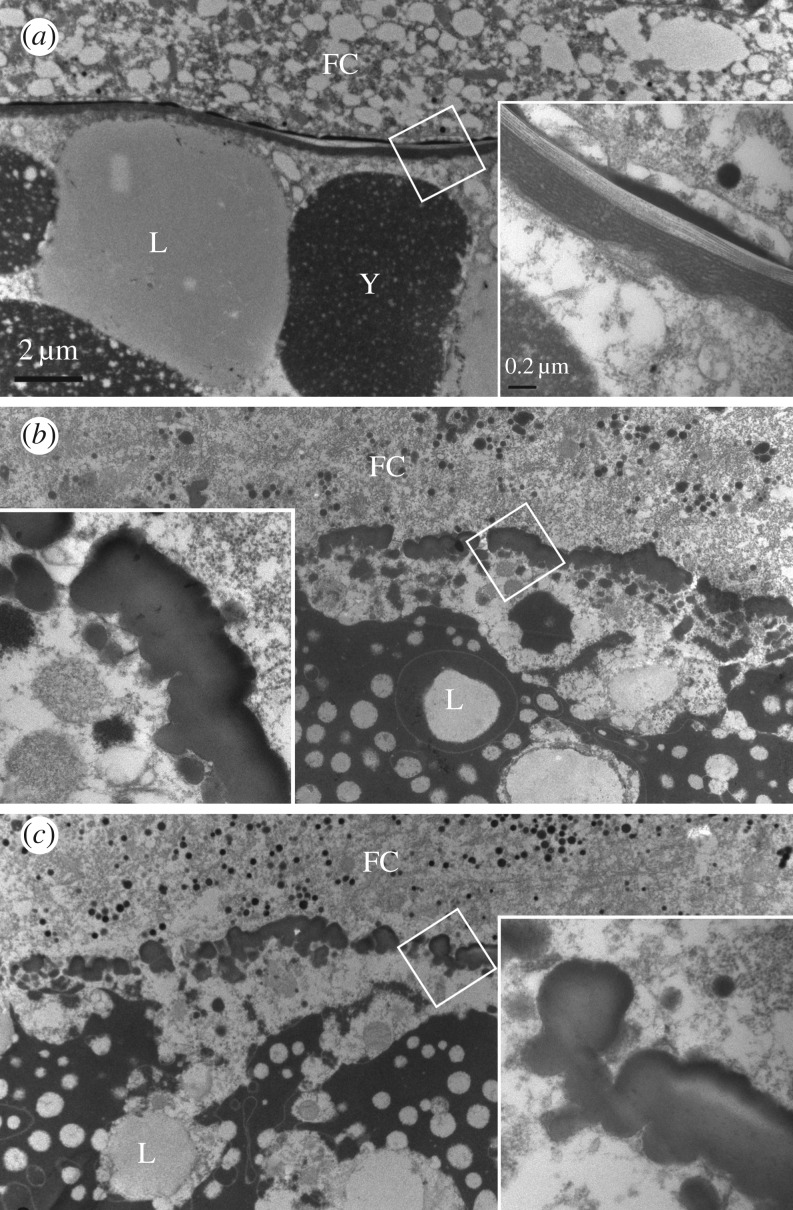
Ultrastructural analysis of oocytes in the basal part of ovarioles. Samples for examining the ultrastructure of ovarioles were prepared by dissecting the ovaries of adult females which had been treated with dsRNA for 5 days. (*a*) For ds*GFP*; (*b*) for ds*NlFoxL2* and (*c*) for ds*NlFcp3C*. The insets show the enlargement of the boxed areas of the chorion. FC, follicular cells; Y, protein yolk spheres; L, lipid droplets.

As oocyte dysplasia was the major contributor to the abnormal phenotype, we further observed the ultrastructural structure of oocytes in the basal egg chamber of ovarioles using a transmission electron microscope (TEM). The ovarioles were prepared by dissecting the ovaries of adult females 5 days after injection. In the ds*GFP* group, the oocytes in the basal egg chamber of ovarioles were surrounded by cells of the follicular epithelium on the outside and filled up with protein yolk spheres and lipid droplets. The chorion, sandwiched between the follicular cells and the oocyte protoplast, had a continuous multilayer structure (figure 7*a*). After knockdown of *NlFoxL2* or *NlFcp3C*, no structural abnormalities of the follicular cells were observed, but the structure of the chorion had substantial aberrations. The chorion became fractured and could not keep the continuous multilayer structure (figure 7*b*,*c*).

**Figure 8.**
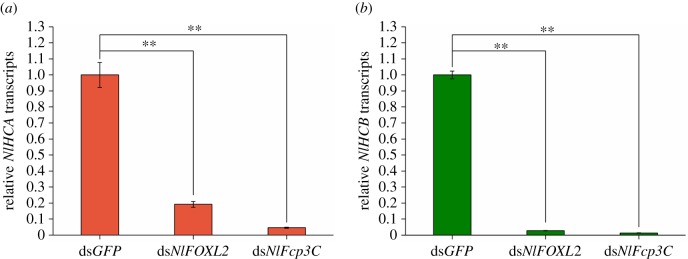
Knockdown of *NlFoxL2* or *NlFcp3C* affects the expression of chorion genes. (*a*) Relative transcripts of *NlHCA*; (*b*) relative transcripts of *NlHCB*. dsRNA (50 ng per insect) for *NlFoxL2* or *NlFcp3C* was injected into newly emerged female adults (within 2 h). RNAi efficiency was investigated using real-time qPCR. Each total RNA sample for these two genes was extracted from 10 BPHs 3 days after injection. ds*GFP* was injected as negative control for the non-specific effects of dsRNA. Values are means ± s.e.m. from three experiments. **p < 0.01 (Student's *t*-test), difference from ds*GFP*.

In further experiments, we found that *NlFoxL2* or *NlFcp3C* depletion suppressed the expression of genes encoding high-cysteine chorion proteins (*NlHCA* and *NlHCB*) (figure 8).

### 2.6. *Nl*FoxL2 activates the *NlFcp3C* promoter

Experimental results of RNAi efficiency by qPCR revealed that *NlFcp3C* is a downstream gene of *NlFoxL2*: knockdown of *NlFoxL2* efficiently suppressed the transcript levels of *NlFcp3C*, but knockdown of *NlFcp3C* did not affect expression levels of *NlFoxL2* (figure 4). Transient expression assays were performed to test whether *Nl*FoxL2 was a direct activator of the *NlFcp3C* promoter.

**Figure 9.**
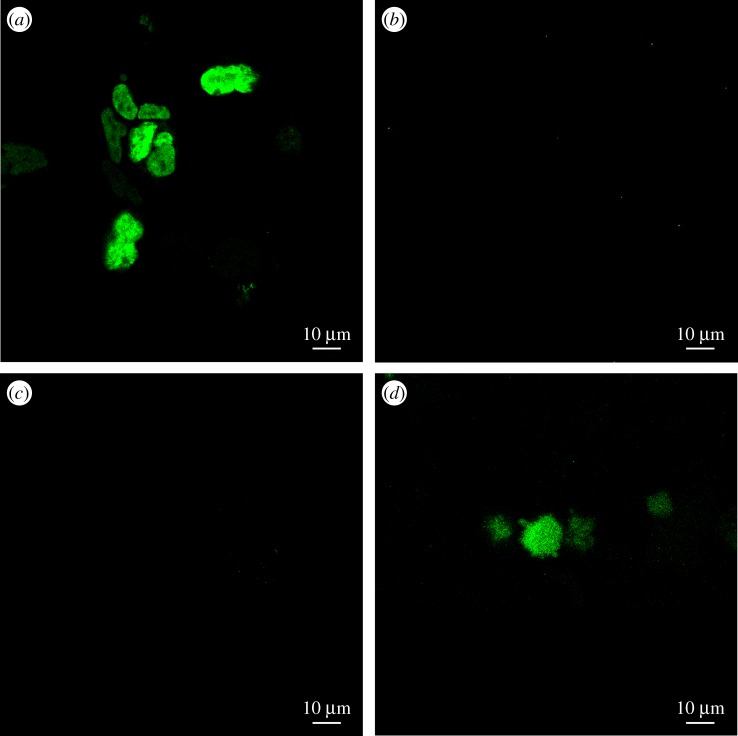
*Nl*FoxL2 activates the *NlFcp3C* promoter. (*a*) HEK-293T cells were transfected with pN1-*NlFoxL2*-*EGFP* as positive control; (*b*) HEK-293T cells were transfected with pN1-*NlFoxL2* as negative control; (*c*) HEK-293T cells were transfected with pT1-prom3C-*EGFP* as negative control; (*d*) HEK-293T cells were co-transfected with pN1-*NlFoxL2* and pT1-prom3C-*EGFP* as an experimental group.

Forty-eight hours after the transfection of HEK-293T cells, an enhanced green fluorescent protein (EGFP) signal was detected. As positive control, HEK-293T cells transfected with pN1-*NlFoxL2*-*EGFP* showed extensive EGFP fluorescence, suggesting that the plasmid pN1-*NlFoxL2*, which had a termination codon (TGA) between the *NlFoxL2* ORF and *EGFP* ORF, could work well in HEK-293T cells to express *Nl*FoxL2. No detectable EGFP signal was observed after transfection with the pN1-*NlFoxL2* or the pT1-prom3C-*EGFP*. By contrast, cells transfected with pT1-prom3C-*EGFP* showed detectable EGFP signal when co-transfected with pN1-*NlFoxL2*. This result demonstrated that *Nl*FoxL2 could activate the *NlFcp3C* promoter to drive EGFP expression in HEK-293T cells (figure 9).

## 3. Discussion

Our analysis showed that the sequences of FoxL2 are highly conserved in vertebrates and invertebrates, and the roles of FoxL2 in ovarian development also seem somewhat conserved. FoxL2 in mammals mainly affects the differentiation of granulosa cells [9], while our results showed that FoxL2 in insects plays a major role in the regulation of chorion formation. This difference could be because mammals are viviparous and do not have chorions, thus leading to the differentiation of FoxL2 target genes. Our research first discovered that *Nl*FoxL2 played an important role in chorion formation by activating *NlFcp3C* directly.

*Fcp3C* was first identified in *Drosophila melanogaster*, encoding a protein involved in the formation of the VM, the first secreted chorion layer [21]. In *Blattella germanica*, the mRNA of *Fcp3C* appeared in 3-day-old females and peaked at choriogenesis [22]. In *Diploptera punctata*, the expression patterns also suggested that *Fcp3C* plays a role in chorion formation [22,23]. Furthermore, failure in forming chorion layers caused by depletion of *Bg*Windei by RNAi was coupled with significant underexpression of *Fcp3C* [24]. However, there is no direct evidence to prove the function of Fcp3C. Our results gave the direct evidence that *Fcp3C* played an important role in chorion formation.

The results determined by qPCR and EGFP assay showed that *Nl*FoxL2 directly targets the expression of *Nl*Fcp3C, which might explain why knockdown of *NlFoxL2* or *NlFcp3C* had similar results. *Nl*FoxL2 plays an important role in the ovaries by regulating the expression of *Nl*Fcp3C. *Nl*Fcp3C depletion suppressed the transcript levels of chorion genes (*NlHCA* and *NlHCB* expressed during late choriogenesis) and prevented chorion formation. Our TEM observations showed that *NlFoxL2-* or *NlFcp3C*-knockdown follicle cells could not form proper chorion layers around the egg. As the abnormal oocytes could not be oviposited, they could only remain stuck in the ovaries. The accumulation of the abnormal oocytes in the ovaries might be the reason for the observed obesity.

In conclusion, our results identified *Nl*FoxL2 as a direct activator of the *NlFcp3C* promoter. Depletion of *NlFcp3C* mRNA by RNAi in *N. lugens* females prevented normal chorion formation. In turn, it impaired oviposition function and eventually prevented reproduction and caused obesity.

## 4. Experimental procedures

### 4.1. Insects

The insects used in this study were obtained from local rice fields at Zhejiang University, Hangzhou, Zhejiang, China. The insects were reared on fresh rice seedlings (Xiushui 134) at 25 ± 1°C and 60–70% relative humidity under a 16 L/8 D photoperiod [25].

### 4.2. Gene identification and sequence analyses

*Nilaparvata lugens* genomic (GenBank accession numbers: AOSB00000000) [26] and transcriptomic databases were screened for genes encoding FoxL2 and Fcp3C against the amino acid sequences from *Drosophila melanogaster*, *Blattella germanica*, *Mus musculus* and *Homo sapiens*, which were obtained from GenBank. The full-length cDNA sequences of the two genes were obtained from transcriptomic databases and confirmed by RT-PCR. Multiple sequence alignments were performed using the ClustalX program [27]. Based on the 5′-untranslated region (5′-UTR) sequence described above, a 1395 bp fragment of the promoter region of *NlFcp3C* was obtained from the genomic database and confirmed by PCR. The primers used here are shown in the electronic supplementary material, table S1.

### 4.3. Total RNA isolation and first-strand cDNA synthesis

Total RNA from whole insects at various developmental stages or from tissue samples was isolated using a TRIzol Total RNA Isolation Kit (Takara, Kyoto, Japan). Developmental samples were collected from different stages of BPHs (*n* = 15–20), including eight egg samples (every 24 h after the eggs were laid), 28 nymph samples (every 24 h after moulting for first and second instars; every 12 h after moulting for third, fourth and fifth instars), 11 female adult samples (every 12 h after moulting) and 11 male adult samples (every 12 h after moulting). Similarly, various tissue samples including integument, gut, Malpighian tube, fat body, salivary gland and ovary were dissected from random female adults 48–72 h after adult emergence. To further characterize the expression in ovaries, the ovaries of female adults 48–60 h after eclosion were dissected and further divided into five parts: oocyte, ooecium wall, ovipositor, terminal filament and fallopian tube. First-strand cDNA was synthesized using a ReverTra Ace qPCR RT Master Mix with gDNA Remover (Toyobo, Osaka, Japan) using 0.5 µg of total RNA template in a 10 μl reaction, following the manufacturer's protocol.

### 4.4. Real-time qRT-PCR analyses

To investigate the developmental and tissue-specific expression patterns, real-time quantitative polymerase chain reaction with reverse transcription (qRT-PCR or qPCR) was conducted using pairs of gene-specific primers designed using the Primer Premier 6 program (electronic supplementary material, table S1) and the cDNA prepared as described. The qPCR reactions (20 µl each) contained 2 µl of cDNA diluted 10-fold, 0.6 µl of each primer and 10 µl of SYBR Premix Ex Taq, and were run in a Bio-Rad Real-time PCR system (Bio-Rad, Hercules, CA, USA). The *N. lugens* housekeeping gene for 18S ribosomal RNA (*Nl18S*) (GenBank accession number JN662398.1) was used as an internal control. The qPCR programme consisted of an initial denaturation step at 95°C for 30 s, followed by 40 cycles at 95°C for 5 s and 60°C for 30 s. A relative quantitative method (^ΔΔ^C_t_) [28] was applied to evaluate the variation in expression among samples.

### 4.5. RNAi effects on *Nilaparvata lugens*

The double-stranded RNA (dsRNA) was synthesized from the purified DNA templates, which were prepared by RT-PCR amplification using a MEGA script T7 Transcription kit (Ambion, Austin, TX, USA). A unique region of each gene was chosen as a template for dsRNA synthesis. The primers used for dsRNA synthesis are shown in the electronic supplementary material, table S1. The dsRNA for *GFP* was used as negative control for the non-specific effects of the dsRNA. Microinjection of planthoppers with dsRNA was carried out according to a method reported previously [29]. Newly emerged female adults (within 2 h) were chosen to be injected. One hundred and fifty insects were used for each gene treatment, and each treatment was conducted in triplicate. Each insect was injected with 10 nl of dsRNA at a concentration of 5 μg µl^−1^. Samples were collected from a set of six to ten insects to evaluate the RNAi effects of each gene (sample time: 3 days after injection).

### 4.6. Determination of BPH weights

The average weight of the female adults (1–5 days after injection) was calculated by weighing BPHs in groups using an AB204-N precision scale (Mettler-Toledo, Ohio, USA).

### 4.7. Transmission electron microscope observations

Samples for examining the ultrastructure of ovarioles were prepared by dissecting the ovaries of adult females which had been treated with dsRNA for 5 days. Sample treatment procedures were carried out according to a method reported previously [30]. Ultrathin sections were examined with a JEX-1230 TEM (JEOL, Tokyo, Japan) at an accelerating voltage of 80 kV.

### 4.8. Promoter assay

The full-length *NlFoxL2* ORF (primers: L2-ATG-XhoI-F, L2-CG-BamHI-R) was cloned into the pEGFP-N1 vector with enhanced green fluorescent protein (EGFP) at the C-terminal end (pN1-*NlFoxL2*-*EGFP*) via the BamHI/XhoI sites to obtain the fusion protein *Nl*FoxL2-EGFP. Similarly, the full-length *NlFoxL2* ORF with a TGA stop codon at the 3′ end (primers: L2-ATG-XhoI-F, L2-TGA-BamHI-R) was cloned into the pEGFP-N1 vector (pN1-*NlFoxL2*). The 1395 bp fragment of the *NlFcp3C* promoter region (primers: prom-3C-F, prom-3C-R) was cloned into the pEASY-T1 vector (TransGen Biotech, Beijing, China) in the forward direction (pT1-prom3C). The *EGFP* ORF was obtained by PCR (primers: EGFP-ATG-NotI-F, EGFP-TAA-XbaI-R) using pEGFP-N1 as a template and inserted in the pT1-prom3C via NotI/XbaI (pT1-prom3C-*EGFP*). Cell culture and transfection protocols were previously described by Yang *et al.* [31]. HEK-293T (human embryonic kidney 293T) cells were co-transfected with pN1-*NlFoxL2* and pT1-prom3C-*EGFP* as an experimental group. pN1-*NlFoxL2*-*EGFP* was transfected into HEK-293 cells as positive control. pN1-*NlFoxL2* or pT1-prom3C-*EGFP* were transfected into HEK-293 T cells as negative control. For the fluorescence microscopy, cells were observed using a Zeiss LSM510 microscope 48 h post-transfection.

## Supplementary Material

Figure S1. Alignment of 17 FoxL2 orthologs; Table S1 Primers used in this work.

## Data accessibility

Sequences of *NlFoxL2* and *NlFcp3C* are available at NCBI, *N. lugens* genome (GenBank accession numbers: AOSB00000000).
