# Supplementary material for: Forkhead box transcription factor L2 activates Fcp3C to regulate insect chorion formation
Source: Open Biol. 2017 Jun 14;7(6):170061. doi: 10.1098/rsob.170061 (PMC5493777; doi:10.1098/rsob.170061)
Supplement: Figure S1. Alignment of 17 FoxL2 orthologs; Table S1 Primers used in this work. [file rsob170061supp1.pdf]

Table S1. Primers used in this work.

Primer usage	Primer name	Primer sequence (5'- 3')
**Synthesis of dsRNA**	T7-L2-F	taatacgactcactatagggCTCTACGCCGACTCGCCCTACCAC
	T7-L2-R	taatacgactcactatagggTCGGCGATTCATCTTTCATTTCTG
	T7-3C-F	taatacgactcactatagggCCGACAACCATGTTTCAC
	T7-3C-R	taatacgactcactatagggTCCATTCTTGCAGCAGTAT
**qPCR**	q-L2-F	GCAGCGGGAGGGCGGAGAT
	q-L2-R	GGGGGCGGCATGGTGGTTGT
	q-3C-F	CCCTGCTATTATCTGTGG
	q-3C-R	ATTTAGGCTATGGGACAC
	q-18S-F	GTAACCCGCTGAACCTCCT
	q-18S-R	TCCGAAGACCTCACTAAATC
	q-HCA-F	GTGAGCAGATTAGGATTGTG
	q-HCA-R	AAGGCGATGAAGAGCAATA
	q-HCB-F	CAGCATCACGATTGACTTC
	q-HCB-R	TACTTCCGCAACAGCATT
**Clone segments**	full-L2-F	GTATTGTGGAGTGGTTGGA
	full-L2-R	GACTGAGATGATCGCTTGA
	full-3C-F	GTTTGAAAGTGGTTGGTGAA
	full-3C-R	GCTCATCAAGAACACATACC
	prom-3C-F	ATGAAGACAGTATTGTGCTG
	prom-3C-R	TTCACCAACCACTTTCAAAC
**Construction of vectors**	L2-ATG-XhoI-F	ctcgagATGGTGTCTCATCTGCTACCAGAC
	L2-CG-BamHI-R	ggatccCGCGACATGAAACACTTTGGTC
	L2-TGA-BamHI-R	ggatccTCACGACATGAAACACTTTGGTC
	EGFP-ATG-NotI-F	gcggccgcATGGTGAGCAAGGGCGAGG
	EGFP-TAA-XbaI-R	tctagaTTACTTGTACAGCTCGTCCATGCC

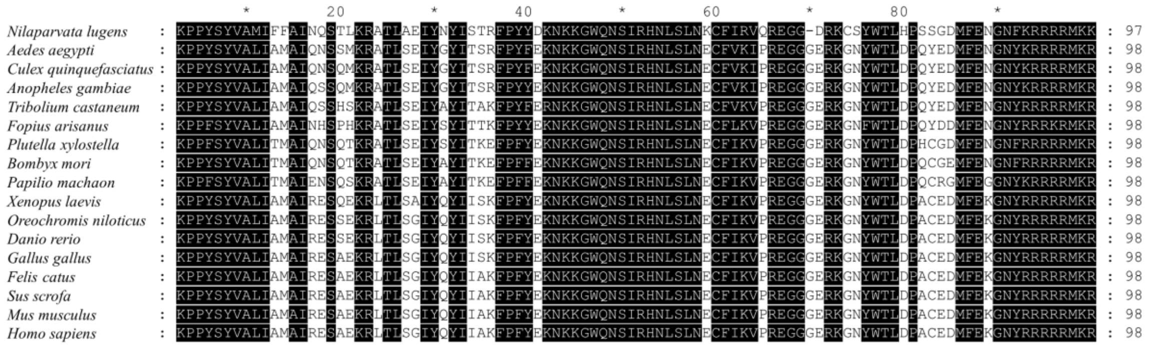

Figure S1. Alignment of 17 FoxL2 orthologs.

*Nl*FoxL2 was aligned with FoxL2 orthologs from 16 other insect species using the ClustalX program.
